# Undoing disparities in faculty workloads: A randomized trial experiment

**DOI:** 10.1371/journal.pone.0207316

**Published:** 2018-12-19

**Authors:** KerryAnn O’Meara, Audrey Jaeger, Joya Misra, Courtney Lennartz, Alexandra Kuvaeva

**Affiliations:** 1 Department of Counseling, Higher Education, and Special Education, University of Maryland, College Park, Maryland, United States of America; 2 Department of Educational Leadership, Policy and Human Development, North Carolina State University, Raleigh, North Carolina, United States of America; 3 Department of Sociology, University of Massachusetts, Amherst, Massachusetts, United States of America; Indiana University, UNITED STATES

## Abstract

We conducted a randomized control study to improve equity in how work is taken up, assigned and rewarded in academic departments. We used a four-part intervention targeting routine work practices, department conditions, and the readiness of faculty to intervene to shape more equitable outcomes over an 18-month period. Our goal was to (a) increase the number of routine work practices that department faculty could enact to ensure equity, (b) enhance conditions within the department known to positively enhance equity, and (c) improve the action readiness of department faculty to ensure equity in division of labor. Post intervention faculty in participating departments were more likely than before the intervention to report work practices and conditions that support equity and action readiness in their department, and that teaching and service work in their department is fair. Participating departments were significantly more likely than control departments to report practices and conditions that support equity and greater action readiness to address issues of workload equity in their department. Finally, participating department faculty were more likely than control department faculty to report increased self-advocacy and were more likely than control department faculty to report that the distribution of teaching and service work in their department is fair.

## Introduction

Across STEM and non-STEM fields, women faculty spend more time on service, undergraduate teaching, and mentoring, while men spend more time per week on research [1–5]. The small numbers of faculty women and faculty from underrepresented minority groups in STEM fields exacerbate unequal and unrecognized service and mentoring loads, especially for women of color [1, 6–8]. Institutional housekeeping and campus service activities are often devalued in academic reward systems [1, 9–11]. Given the importance of research products, funding, and visibility for advancement in STEM fields, spending less time on research, and more on service, teaching, and mentoring is especially problematic. Systemic inequities in workload have been identified as central to STEM women’s lower tenure and promotion rates, longer time to promotion to full professor, and greater career dissatisfaction [1, 12, 13].

The consequences of organizational dynamics that constrain faculty workload and rewards in academic careers are significant, particularly for STEM faculty women and members of underrepresented groups. The conditions in most academic departments where teaching and service work is taken up, assigned, and rewarded among members make this challenge seem intractable. Rather than a single pivotal decision, disparities in faculty workload are the result of a series of many decisions being made in departments where the division of labor is also changing and evolving over time. To address this challenge, we designed an intervention aimed at creating greater workload equity within departments. This randomized experiment provides evidence that it is possible to create fairer faculty workloads.

Research in behavioral economics and social psychology on diverse populations worldwide spotlights the irrational, biased, and unconscious way people tend to make decisions, but also how such limited thinking can be disrupted or reshaped through behavioral design or “nudges” [14–18]. The challenge for higher education institutions is to apply the lessons learned from this behavioral work to reduce biases as they might appear in the thorniest academic spaces and situations, such as the division of labor in academic departments.

Behavioral design research explains how to redesign the “choice architecture” around important decisions by changing the context within which people make decisions [18]. For example, common tools in choice architecture are changing the order in which options are presented, setting a more desirable default option, framing the decision differently, providing information or feedback, and creating incentives [19]. Iris Bohnet observes “there is no design free world” [14]. How we currently make decisions, including how we divide collective work in departments, is not neutral. Organizational members must decide how to assign and reward work critical to the department that is not particularly desired or advantageous to academic careers. Given the likelihood of inequitable workloads, “why not design a bit more thoughtfully?” [14].

We drew on choice architecture to guide a randomized control study aimed at improving equity in how work is taken up, assigned, and rewarded in STEM academic departments. Our study employed the National Science Foundation definition of STEM [20] which includes mathematics, natural sciences, engineering, computer and information sciences, and the social and behavioral sciences–social psychology, economics, anthropology, sociology, and political science. As such, we delimited our work to focus only on departments that NSF defines as STEM, rather than a more diverse group of departments.

Our intervention targeted routine work practices, department conditions, and the readiness of faculty to intervene to shape more equitable outcomes. Routine work practices might be thought of as default valves or levers of the machine that order the “choice architecture” of how work is taken up [19]. Department conditions are the backdrop of assumptions, priorities, knowledge, and informal operating procedures that shape workload allocation. Action readiness is the degree to which faculty in a department feel they are able and willing to act to ensure fairness in equitable workload allocation [21–23].

We aimed to improve: transparency in what faculty are doing, accountability, clarity in roles and expectations, and flexibility to acknowledge different contexts. Transparency increases sense of accountability and trust between members and leaders, facilitates perceptions of procedural and distributive justice, and leads to greater organizational commitment [24–27]. Departments that routinely make data on faculty activities accessible are likely to promote perceptions that workloads are transparent and fair [28].

In addition, research shows that inequity and biases operate more in environments with ambiguous evaluation criteria [29–31]. Women and members of URM groups are more likely to be disadvantaged when standards for faculty evaluation are “foggy” [32, 33], not receiving the same benefit of doubt with regard to performance that groups in the majority receive. Alternatively, environments with concrete, objective evaluation criteria, “mitigate the operation of prejudices” and inequity [33]. Clear criteria, uniformly applied, enhance confidence in procedural and distributive justice [25]. Thus, departments with clear benchmarks for performance and accountability for meeting them are likely to see more equitable workloads.

Equity-minded departments often have shared rotation of time intensive, less promotable, but necessary work, as well as rotation of more preferred roles. Faculty do not volunteer, or opt in; rather, they have to opt out, which is more difficult to do. This ensures that everyone does their fair share of a group’s collective work, facilitating equity norms, social responsibility norms, and norms of reciprocity [34]. Planned rotations send the message that everyone has to chip in and help avoid “free-riding” wherein one group member or more fail to do their fair share of the work and others compensate [35–37]. Such practices can change the conversation from, “why would I agree to do that?” to “how can I argue that I alone should not have to do this?” Such practices facilitate more equitable workloads in departments.

Finally, equitable systems acknowledge differences in contexts [38]. Faculty work under structural, social, and cultural contexts which make experiences and workloads distinctly different. Teaching the department's only service-learning course and supervising students in community placements may be more time intensive than teaching a large lecture with TAs. Reward systems can either recognize such differences by using modified workload plans, or make them invisible [39]. Rousseau found that personalized employment arrangements are often an important part of equity and acknowledging difference [40]. However, co-worker acceptance of these deals can affect these arrangements. Departments interested in fairly dividing different kinds of work need to develop well-established benchmarks and procedures to ensure employees recognize these arrangements as reflecting procedural, interactional and distributive justice [41].

We designed a set of interventions, informed by research on choice architecture, to enhance department workload equity. We hypothesized that academic departments randomly assigned to our interventions would see department conditions, work practices, and action readiness among department members increase, compared to departments with no intervention. Theory driven randomized control trials with faculty as participants are rare in higher education research [42], and we are not aware of any study like ours focused on faculty workload equity specifically, though other studies have tried to shape more equitable workloads as part of overall department climate [43–46].

We designed and empirically tested over an 18-month period a four-part intervention aimed at improving faculty experience of workload equity in STEM academic departments. This randomized control trial was meant to understand the efficacy of our intervention to improve faculty satisfaction and experience of equitable workloads. Control-treatment studies are the gold standard in determining whether interventions are effective [47]. The few studies that have been done to assess the efficacy of diversity related interventions with higher education faculty tested interventions aimed at improving inclusive hiring practices [48–50] and they likewise used a control-treatment method to ensure confidence that their interventions were efficacious and should be replicated.

A call for participation went out to all provosts and STEM department chairs at four-year public institutions in Maryland, North Carolina, and Massachusetts. These states were selected based on proximity to the Project PI’s and project leadership. Thirty academic departments from a range of different institutional types were then enlisted in the research study. Our goal was to (a) increase the number of routine work practices that department faculty could enact to ensure equity, (b) enhance conditions within the department known to positively enhance equity, and (c) improve the action readiness of department faculty for ensuring equity in division of labor. Interventions are discussed in more detail in supplementary materials. They included a workshop on how implicit bias can shape faculty workload allocation, guidance to collect and share transparent annual faculty work activity data (a “dashboard”), showing how the dashboard could identify equity issues, providing a variety of sample organizational practices that address equity issues, and department development of a “Department Equity Action Plan,” adopting organizational practices that they thought would solve the equity issues their dashboards had revealed. In addition, faculty members took part in an optional 4-week individual time management and planning webinar.

The research questions guiding this study were: Do departments that participate in an intervention to improve equitable department workloads report stronger department work practices, conditions, action readiness, and greater fairness in workload pre to post intervention? Do departments that participate in an intervention to improve equitable department workloads report stronger department work practices, conditions, action readiness, and greater fairness in workload than matched control departments?

## Materials and methods

We designed a cross sectional survey to collect needed data to examine our research questions, compare control and participating departments to each other, and understand the influence of the intervention in participating departments over time (Table 1 describes measures). We considered the ethical implications of withholding treatment to the control group but felt it was appropriate for two reasons. First, although there was good evidence in our literature review that these 4 interventions would be successful in shaping workload equity there was no previous study to prove that was the case as this is the first study of its kind. As such, we were not withholding a proven treatment. Second, we made an agreement with control departments that they would receive all of the tools and resources from the project that the treatment departments received at the project conclusion and that they could participate with us in a subsequent implementation.

**Table 1:** Survey items descriptive statistics.

Constructs	Survey Item	Post-Survey		% Agree/ Strongly Agree		Standardized item loading
		Mean	Std. Dev.	Pre-Survey	Post- Survey	
Department Conditions	There is awareness of implicit bias	2.04	.80	20.9	35.3	.753
	There is a commitment that workload be fair	2.48	.75	24.2	64.6	.787
	The most important work is credited	2.30	.86	28.5	60.6	.842
Work Practices	Transparent work activity data is published	2.04	.85	45.8	63.5	—
Action Readiness	Faculty know strategies to improve fairness	3.60	1.28	41.5	59.1	.798
	Faculty have concrete steps to ensure equity	3.37	1.32	37.2	49.5	.852
	Faculty can use data to initiate discussions about workload	2.92	1.26	57.7	63.8	.507
	Faculty can create benchmarks for work activities	3.11	1.23	68.5	71.3	.642
Perception of Fairness	Distribution of teaching and service work is fair overall	2.09	.70	72.8	77.2	—
Self-Advocacy	Faculty feel they can say no to requests	3.35	1.05	41.0	50.8	.900
	Faculty feel comfortable protecting time	3.86	1.37	47.4	65.3	.698
	Faculty feel comfortable asking for additional resources	3.70	1.37	42.6	59.9	.841

*Pre- to post- percent is based on pre- to post- matched respondents

The survey was approved by the University of Maryland Institutional Review Board (Approval number [738322–3] Faculty Workload and Rewards) and participants signed an electronic informed consent form before completing the survey. Although measuring changes in actual workload over time would have been revealed important data about the efficacy of the interventions, such changes can take several years to emerge and appear small in departments with very low numbers of faculty. Given that faculty perceptions of workload equity are associated with overall faculty satisfaction and intent to leave [51,52] self-reported measures and experiences are also an important measure and appropriate way to see if interventions have had intended effects [22].

The *work practices* and *perception of fairness* were analyzed as single-item constructs. The *work practices* construct captures the presence or lack of organizational practices such as transparency of data. *Department conditions* were measured as having awareness of biases, workload fairness and work recognition. *Action readiness* was operationalized as knowing strategies and taking action to ensure fairness and equity, and the extent to which department faculty were confident about using workload data and creating more transparent benchmarks. Self-advocacy was assessed as having the confidence in protecting faculty time, asking for resources and saying no to requests. These items were measured using a 5-point Likert-type response scale (e.g. 1-strongly disagree, 2-disagree, 3-neither agree nor disagree, 4-agree 5-strongly agree), and the mean of the items was used as the overall measure of the constructs. We analyzed the variables of gender (male = 0, female = 1), race (White = 0, Faculty of Color = 1), rank (dummy coded with assistant professors as the referent group), and discipline (dummy coded with natural sciences as the referent group).

We received 30 applications from 16 institutions: one baccalaureate institution, six masters, and nine doctoral/research institutions. All departments completed the pre-survey. Pre-survey invitations were sent out to 658 faculty. Of 658 invited faculty, 70.5% (n = 464) responded to the pre-survey. Due to the diversity of the departments in the study and our desire to have fair comparisons, we created matched pairs prior to random assignment, to take into account the key potentially confounding characteristics. Each department in each pairing had an equal chance of being randomly assigned to receive the treatment. After accounting for geographic location, a logistic requirement to facilitate delivery of the intervention, departments were matched on four key characteristics, which included: a) whether the department was at a Doctoral granting/Research institution, a Master’s granting institution, or a Baccalaureate granting institution, b) whether the department was in the Natural Sciences or Social Sciences disciplines, c) whether the department was small, medium, or large in size (0–15, 16–30, and 31–60, correspondingly), and d) whether the representation of women faculty was low, medium, or high (1–34%, 35–50%, and 51–100%, correspondingly).

As institutional type and disciplinary group were considered the most important characteristics, all pairings had to match at least on these two. Likewise, size of department was determined to be a key characteristic and the majority of pairings were matched on it. Presence of women, while important, was determined to be less potentially confounding so was less important in matching the pairs. In order to balance geographical constraints we needed to allow four more departments into the study as participating, we added them to pairs where their characteristics matched. Generally, one department from each matched set was randomly selected to participate in the treatment. However because of the matching criteria listed above, there were four treatment departments that had to be matched with an already matched control department. This led to 17 participating and 13 control departments total.

We worked with the 17 participating departments for 18 months on the four interventions. Each department created a team of between 3–5 members. Although department teams were the primary participants in interventions, all of the information shared in workshops and with department teams were also made available to all members of departments. The teams regularly updated their departments on what they were gaining and planning. We met department teams for ½ day workshops four times over those 18 months and held several check-in calls between those meetings. During the first workshop we provided an experiential workshop on implicit bias and the research on how it shapes divisions of labor in colleges and universities. We also shared aggregate reports with department teams of their pre-survey data. During the second workshop we provided department teams training, tools and resources to create their work activity dashboards. During the third workshop we shared evidence-based policies and practices departments could use to proactively shape equitable workloads. Examples include such polices as credit systems, rotation policies for time intensive roles and differentiated workload policies. Department teams then began creating Department Equity Action Plans (DEAP), which were 2 page descriptions of the data they had reviewed from their own department, the equity issues they wanted to address, and the policies and practices they would put in place (pending consensus from their departments) to ensure greater equity moving forward. Over the course of the following months the teams worked with project leadership to further refine their DEAPs over monthly check-in calls. During a fourth workshop departments from all 3 states shared their DEAP’s with each other in a final capstone event. In addition, treatment department faculty members took part in an optional 4-week individual time management and planning webinar which was for their own professional development, not connected to the other three initiatives.

All departments were represented at each of the four interventions. There were a few transitions of individuals on and off teams because of leadership changes, parental leaves or illness. However, all departments received all 4 interventions. These four interventions were intended to work synergistically to improve conditions, practices, action readiness and perceptions of workload fairness. Treatment departments were asked to keep all project materials within their department.

Researchers had no contact with control departments after the pre-survey was completed. At the completion of the project, we sent a post-survey to control and participating departments. We sent 635 post-survey invitations to department faculty and 472 agreed to participate (demographic characteristics of the sample can be found in Table 2). Some faculty members had retired or left their departments; others had been hired over this period. The post-survey response rate was 74.3%; we matched 326 participants (69% of all respondents) from control and participating departments who took both pre and post surveys.

**Table 2:** Respondent demographics, post-survey.

Rank	Assistant Professors	21.2%
	Associate Professors	29.8%
	Full Professors	28.9%
	Non Tenure-Track Faculty	20.1%
	American Indian or Alaska Native	0.7%
Race	Asian	9.6%
	Black/African American	10.7%
	White	75.1%
	Multi-Racial	3.9%
Gender	Female	45.8%
	Male	52.5%
	Other	1.8%

**Fig 1:**
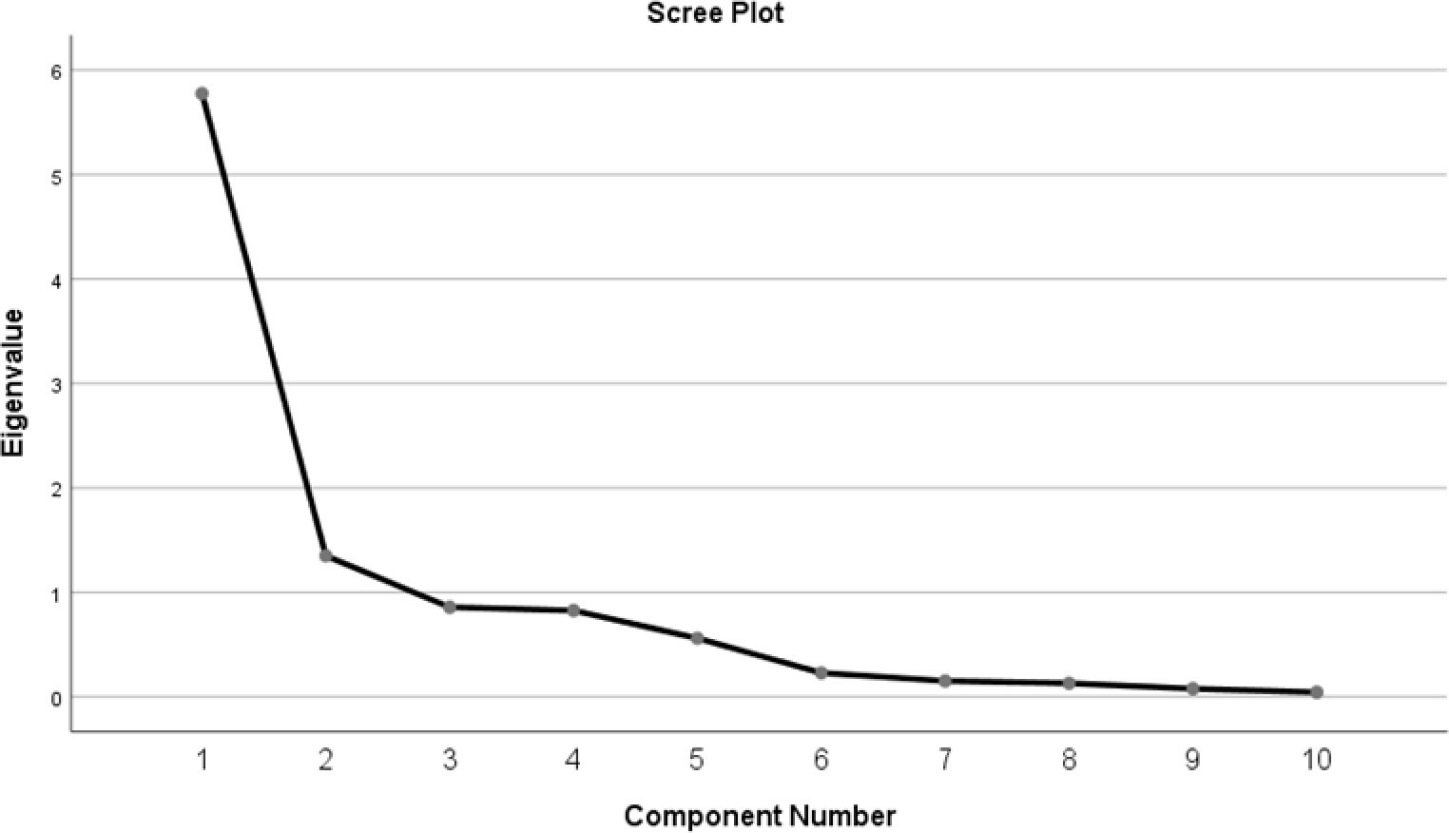
Cattell’s scree plot of eigenvalues in PCA analysis.

First, to reduce the data into larger composites we conducted Principal Component Analysis (PCA) using oblique (non-orthogonal) factor rotation method (Direct Oblimin with Kaiser Normalization). Based on Cattell’s scree plot (Fig 1.), Kaiser-Guttman rule of eigenvalues greater than one and item loadings in pattern matrix we extracted three factors in addition to the two single item constructs of perception of fairness and work practices: department conditions, action readiness, and self-advocacy. We tested the construct validity of the identified latent factors using Confirmatory Factor Analysis (CFA) [53]. We retained items with standardized loadings of 0.5 and higher (Table 1). For descriptive purposes Table 1 also includes means and change in pre- to post- survey responses on the selected items.

Next, we ran regression analyses on the determined factors controlling for gender, race, rank, discipline and interaction of gender and race. For the purposes of verification of regression results we conducted Hierarchical Linear Modeling (HLM) that accounted for department clustering of the data. As outcome variables, we used department conditions, work practices, action readiness, perception of fairness and self-advocacy. As level-1 predictors we used group-centered variables of gender, race and rank. As level-2 predictors we used discipline, department size and gender composition in the department. The fully unconditional HLM model is presented below:

Level-1 Model: *Y_ij_* = *β_0j_* + *r_ij_*
Level-2 Model: *β_0j_* = *γ_00_* + *u_0j_*

The model specifies that a survey response score *Y*_*ij*_ of a faculty member *i* in department *j* is a function of the mean response score across departments *γ*_*00*,_ the random effect of department *u*_*0j*_ (variation between departments), and the random effect of a faculty member *r*_*ij*_ (individual variation).

## Results

### Differences between participating and control departments over time

Comparing participating to control departments, we have clear evidence that the interventions made a difference (Table 3). Controlling for gender, race, interaction of gender and race, discipline and rank, regression analyses on change scores in pre- to post- matched responses showed that participation in project activities was a significant, positive predictor of equitable work practices, action readiness, perception of fairness and self-advocacy, though not on department conditions. For example, participating white women (*Beta* = .199) and minority women (*Beta* = .142) faculty, post intervention, were more likely than control groups of white women and minority women non-participants to report equitable work practices being in place in their departments. Participating white men (*Beta* = .148), white women (*Beta* = .359), and minority men (*Beta* = .148) faculty, post intervention, were more likely than control department faculty to report having increased action readiness. Participating white men (*Beta* = .163), white women (*Beta* = .224), minority men (*Beta* = .155), and minority women (*Beta* = .124) faculty, post intervention, were more likely than control department faculty of the same groups to perceive the distribution of teaching and service work in their department as fair. Participating white men (*Beta* = .143), white women (*Beta* = .159), and minority men (*Beta* = .148) faculty, post intervention, were more likely than control department faculty of the same groups to report an increase in self-advocacy. Interestingly, minority women faculty did not experience increased perceptions of action readiness and self-advocacy after the treatment (Table 3).

**Table 3:** Results from multiple linear regression models, effect of participation pre- to post- change scores, matched respondents, by constructs.

Variable	Department Conditions			Work Practices			Action Readiness			Perception of Fairness			Self- Advocacy		
	Beta	SE	*p*-value	Beta	SE	*p*-value	Beta	SE	*p*-value	Beta	SE	*p*-value	Beta	SE	*p*-value
White Men	.018	.118	.783	.053	.144	.457	.148	.157	.026	.163	.104	.012	.143	.158	.038
White Women	-.012	.120	.851	.199	.147	.004	.359	.156	< .001	.224	.107	< .001	.159	.160	.016
Minority Men	.018	.220	.755	-.041	.271	.526	.148	.285	.013	.155	.189	.008	.148	.300	.017
Minority Women	-.143	.184	.018	.142	.229	.032	.026	.250	.670	.124	.168	.035	-.023	.255	.714
Associate	.003	.126	.964	-.042	.166	.635	-.104	.168	.169	.157	.113	.033	.024	.170	.761
Full	.176	.129	.020	.092	.166	.303	-.050	.171	.513	.191	.115	.011	.090	.175	.260
Non Tenure-Track	.095	.163	.148	-.085	.206	.266	-.173	.210	.011	-.012	.145	.856	.026	.221	.709
Natural Sciences	.025	.100	.668	-.040	.121	.532	.037	.131	.523	.031	.089	.593	.040	.133	.513
Adjusted *R*^2^	.034			.041			.114			.076			.030		

*Note*: Assistant professors and non-participants are referent groups

Overall, participating department faculty, post intervention, were more likely than control department faculty to report transparent information about faculty work activities for all department faculty to see in their department (*Beta* = .138). Participating department faculty, post intervention, were also more likely than control department faculty to report having a good understanding of implicit bias and how it shapes faculty workload (*Beta* = .142). Post intervention, participating department faculty were also more likely than control department faculty to report multiple measures of *action readiness* to address issues of workload equity in their department, such as strategies they can use to improve the perception and reality of fairness in how work is assigned, taken up, and rewarded in their department (*Beta* = .165), having identified several concrete steps they can take to ensure greater equity in their department workload (*Beta* = .181), using data to initiate a dialogue within their department about putting practices in place to ensure the teaching and campus service burden is shared by all (*Beta* = .251), and working with colleagues to create more transparent benchmarks such as advising loads and committee assignments (*Beta* = .142). Participating department faculty were also more likely to report increased self-advocacy such as being able to say no to additional requests (*Beta* = .189), and feeling comfortable asking for additional resources (*Beta* = .133). Participating department faculty were more likely than control department faculty to report that the distribution of teaching and service work in their department is fair overall (*Beta* = .228) (Table 4).

**Table 4:** Effect of participation on pre- to post- change scores, matched respondents.

Constructs	Survey Item	Standardized regression coefficients
Department Conditions		-.011
	There is awareness of implicit bias	.142*
Work Practices	Transparent work activity data is published	.138*
Action Readiness		.265***
	Faculty know strategies to improve fairness	.165**
	Faculty have concrete steps to ensure equity	.181**
	Faculty can use data to initiate discussions about workload	.251***
	Faculty can create benchmarks for work activities	.142*
Perception of Fairness	Distribution of teaching and service work is fair overall	.228***
Self-Advocacy		.164**
	Faculty feel they can say no to requests	.189**
	Faculty feel comfortable asking for additional resources	.133*

Regression analysis was performed on survey items and constructs controlling for gender, race, rank, and discipline. Significant items at *p < .05. **p < .01. ***p < .001.

Table 5 provides descriptive statistics for interaction of gender and race included in the regression analysis.

**Table 5:** Change in means for pre- to post- matched participating faculty.

Constructs	Survey Item	Women		Men	
		White	Minorities	White	Minorities
Department Conditions	There is awareness of implicit bias	.29	.21	.26	.20
	There is a commitment that workload be fair	.63	.21	.76	.93
	The most important work is credited	.54*	-.25*	.59	.53
Work Practices	Transparent work activity data is published	.45	.37	.16	-.17
Action Readiness	Faculty know strategies to improve fairness	.94**	< .01**	.40	.93
	Faculty have concrete steps to ensure equity	1.03*	.19*	.40	.93
	Faculty can use data to initiate discussions about workload	.78*	-.10*	.39	.71
	Faculty can create benchmarks for work activities	.36	.45	.08	.21
Perception of Fairness	Distribution of teaching and service work is fair overall	.22	.18	.16	.37
Self-Advocacy	Faculty feel they can say no to requests	.37	.09	.43	.33
	Faculty feel comfortable protecting time	.59	.14	.34	.86
	Faculty feel comfortable asking for additional resources	.71	.20	.70	1.07

Significant items at *p < .05. **p < .01. ***p < .001.

HLM analysis showed that participation in the intervention was a significant positive predictor of evaluation of equitable department practices (*γ*_*01*_ = .303, SE = .127, *p* = .025), perception of fairness in workload distribution (*γ*_*01*_ = .326, SE = .123, *p* = .013), and action readiness (*γ*_*01*_ = .622, SE = .150, *p* < .001) (Table 6). Gender, race and rank did not have significant fixed or random effects meaning that they did not contribute to department variation in the outcomes.

**Table 6:** Results from final 2-level HLM models.

Variable	Department Work Practices			Action Readiness			Perception of Fairness		
	Coefficient	S.E.	*p*-value	Coefficient	S.E.	*p*-value	Coefficient	S.E.	*p*-value
*Fixed effects*									
GENDER, *γ*_*10*_	.243	.152	.120	.133	.152	.389	-.008	.103	.938
RACE, *γ*_*20*_	-.061	.193	.754	-.149	.290	.611	-.126	.154	.419
PARTICIP, *γ*_*01*_	.303	.127	< .025	.622	.150	< .001	.326	.123	.013
*Random effects*									
	Std. Dev.	Variance Component	*p*-value	Std. Dev.	Variance Component	*p*-value	Std. Dev.	Variance Component	*p*-value
GENDER slope, *u*_*1*_	.355	.126	.059	.152	.023	.187	.058	.003	.175
RACE slope, *u*_*2*_	.038	.001	>.500	.835	.698	.079	.121	.015	>.500
Variance within departments, *r*	.874	.764		.978	.956		.692	.479	

## Discussion

We tested a theory-driven intervention that involved (a) a workshop on implicit bias and how it can shape divisions of labor, (b) arming department teams with tools to create and display faculty workload activity dashboards, (c) using dashboards to identify equity issues and sharing work practices and policies to mitigate bias and proactively design for equity, and (d) an optional professional development webinar series on aligning time and priorities as a faculty member. At the conclusion of this 18-month project, the intervention measurably improved one work practice associated with workload satisfaction—having transparent data on faculty work activities available for department faculty, and likewise improved several conditions related to workload equity such as awareness of implicit bias and commitment among faculty to work being fair. The intervention also improved participating members’ action readiness for ensuring equity in divisions of labor. We believe there was a spillover effect from department member’s putting the a transparent dashboard in place. In other words, as participants saw members of their department were serious about improving equity in division of labor, and recognized their workload relative to others due to the transparent dashboards, they felt greater permission to likewise self-advocate and take steps to ensure their own workload was fair.

There were four limitations. When creating dashboards, project leaders allowed some variation in the levels of transparency provided at the request of faculty and department leaders. As such, some department faculty were potentially “treated” with more or less transparency related to the work activity of colleagues. However, this was mitigated by the fact that all departments were required to provide data in such a form as to allow department members to be able to benchmark their own effort against others, and to see the range of activity within the department. In other words, all treated departments created a basic level of transparency that had not been present before. Second, levels of commitment to the project varied; some departments had five faculty on teams, others had three. There were leadership transitions and unanticipated absences on some teams due to illness, parental leave, and retirements. It is also worth noting that the *optional* time management activity materials were made available to members of all participating departments and many participating members shared materials and discussions with colleagues in the department. However, engagement varied across the participating departments in levels of participation in this one activity. Third, the process of discussing equity issues sometimes evoked negative experiences among colleagues, due to generational differences in the faculty role or frustration that implementing policy reforms took time.

Fourth, the intent of this project was to compare departments that received the 4 treatments to control departments that did not. As such we measured experiences before the project began and shortly after it ended. We do not have data on participants and their behaviors, or conditions several years after the intervention was complete, although we intend to collect another round of data. However, this diversity intervention was in operation 1.5 years, much longer than most diversity interventions which often are measured in a single day at the beginning and end of a workshop and typically try to measure gains in knowledge or attitude [22]. Mechanisms that impact department faculty experience of conditions, practices and action readiness for workload equity are complex. Eighteen months was not long enough for our participating department faculty to experience all potential benefits of new work practices and conditions. When we ended the study, some work practices and policies were just being adopted. Interventions that had more time to take root in departments, such as the implicit bias workshop and dashboard, had the greatest impact. As such, our future research will explore the impact of all four interventions after more time has passed. Likewise, subsequent implementations will tease apart the efficacy of each of the 4 interventions as opposed to examining the effects of all four together on outcomes.

Challenges notwithstanding, our 18-month intervention was successful. We are the only intervention that we are aware of that specifically attempted to and succeeded in changing aspects of the choice architecture of how academic departments allocate workload. One unanticipated finding is that the process of collecting transparent workload data, examining different policy and practice options, and communicating a desire for equity may signal to faculty that others care about equity issues, and in and of itself increase some aspects of satisfaction with workload, independent of actually putting new work practices in place.

Although this study focused on the impact of the four interventions together on outcomes, not the relative value of one or the other, and the importance of their order, there have been a number of other equity and diversity minded interventions that began by having departments collect data together as a necessary precursor to and readying the ground for more concrete policy and practice changes [54]. In addition, efforts to initiate policies and practices to shape workload equity depended heavily on having good data to rationalize policy changes. As such, we believe there is evidence, supported by the literature, to support the order of at least the three main interventions (e.g. implicit bias training, creation of work activity dashboards, and putting in place policies and practices) as best implemented in this order. We will explore this, and other factors such as the value of department chair leadership, in future research.

A diverse faculty is the focus of funding agencies and governments across the world. While much of this effort has involved strengthening the pipeline to scientific careers [55], hiring [50], and interventions to increase awareness of implicit bias and how it affects academic careers more generally [23, 56], increasingly focus has been on retention of women and underrepresented minority faculty members [57, 58]. Departments are at the center of retention efforts because departments are where faculty are hired, take up or are assigned work, and are rewarded. Most decisions to leave academe and institutions can be traced back to experiences within academic departments [59, 60]. Data from national surveys and exit interviews repeatedly show women and underrepresented minority faculty dissatisfied with workload [5, 51, 61] and facing negative career consequences as a result of differential allocation of time to teaching, mentoring, research, and campus service [1, 12, 13]. Our findings contribute tangibly to efforts to understand and change divisions of labor to be more equitable. Departments where faculty experience workload as fair are likely to be places where all faculty are better retained, satisfied, and productive.

## Supporting information

S1 Text Supporting information. (DOCX)

S1 Table Survey items descriptive statistics, all post-survey respondents. (DOCX)

S2 Table Respondent demographics, 2018. (DOCX)

S3 Table Mean differences between pre- and post-survey data, matched faculty participating in intervention. (DOCX)

S4 Table Regression results on change scores for participating vs. control departments, pre- to post- matched respondents. (DOCX)
