# Supplementary material for: Undoing disparities in faculty workloads: A randomized trial experiment
Source: PLoS One. 2018 Dec 19;13(12):e0207316. doi: 10.1371/journal.pone.0207316 (PMC6300212; doi:10.1371/journal.pone.0207316)
Supplement: S1 Text — (DOCX) [file pone.0207316.s001.docx]

**Supporting information**

**Method**

**Interventions**

We designed a four-part intervention to accomplish these goals within academic departments within an 18 month period. We conducted a randomized control trial to understand the efficacy of our intervention to improve faculty satisfaction and experience of equitable workloads. We solicited applications from STEM academic departments in 4 year public colleges and universities in 3 states, NC, MA and MD. To encourage and solicit applications we visited provosts, deans and department chairs and made presentations to their faculty, describing the aims of the project and the interventions that departments would engage if selected as participating departments. We noted that participating departments would receive a stipend of $5,000 to support their work on the project, and control departments would receive a stipend of $1,000. We also made clear that those departments randomly assigned as control departments would receive intervention materials when the research project was over. The project was described as an action research project, approved by the lead author’s institutional IRB Board.

The first part was a three hour workshop on the presence of implicit bias and how it can shape faculty workload allocation. The workshop was modeled after similar workshops that have been implemented and tested on the role of implicit bias in academic hiring [1,2] or implicit bias in academic careers more generally [3,4]. We began the workshop by presenting on the concept of implicit bias and how it has been found through social psychological experiments to shape many aspects of academic careers, including hiring, letters of recommendation, nomination of faculty for awards and teaching evaluations. We then focused on the empirical research on how academic work is taken up, assigned and rewarded specifically, sharing abstracts from social science and education journals showing significant differences in the work of women and men faculty and white and faculty or color in teaching, mentoring, and committee service while controlling for relevant characteristics such as institutional type, gender, race, career stage. We provided a simulation showing how seemingly small differences in workload such as 1.5 hours per week can lead to major differences in time spent on research over a tenure track career. We engaged participants in 2 case study exercises in small groups to experience how workload may be shaped by social role expectations and aspects of identity. We then shared aggregate reports of the pre-survey data with the department teams so that they could examine their own workload and perceptions of department workload practices and conditions. The explicit goal of this intervention was to increase department faculty awareness of implicit bias in how work is taken up, assigned and rewarded. Previous research [1,3] has shown that one of the first steps in mitigating implicit bias is to increase the ability of individuals to see it so that they might intervene later when they see it operating.

The second part of the intervention provided support to departments as they collected annual faculty work activity data. Department teams were provided a template and guidance as to the kinds of data that should be included in data tables and how it might be analyzed (e.g. using descriptive statistics and providing ranges, means, within the department). Departments were encouraged to use extant data as is typically collected in annual faculty reports and sent to department chairs. In most cases the data was readily available, in some cases department teams had to request it from institutional research offices, dean’s offices or similar offices. For example, departments collected how many advisees each full-time faculty member in their department advised and at what level (undergraduate, masters, doctoral). Ultimately department teams created tables or visuals we called “dashboards” showing, for example, that the average assistant professor in the department had 2 doctoral students, 4 master’s students, and 10 undergraduates. Departments were required to analyze by categories of rank and gender when the N within the department of that category was greater than 5, but not otherwise. Departments were allowed to customize their dashboard based on local faculty activities but all included teaching, mentoring, campus service, and administrative activities. Departments submitted their dashboards for feedback to the researchers and received feedback on ways to improve the display of data, differentiate between activities that took more or less time, and achieve the level of transparency and accountability desired. Once completed, about 6 months into the project, departments were required to post their faculty work activity dashboard for all full time faculty to see internal to the university. This intervention had several goals. First, departments were creating a routine work practice that if updated annually, could ensure the condition of greater transparency and accountability for fair divisions of labor. Second, the process of creating the dashboard and later analyzing it enhanced department member’s self-efficacy to use data to make decisions and hold each other accountable. Third, the process of creating the dashboard engaged departments in the process of creating minimum performance standards, differentiating some activities that required more work from others that required less, considering how to “credit” often invisible work, and considering ways in which differentiated workloads could be “fair.”

The third part of the intervention involved working with departments to study their dashboard data to identify equity issues. We used case studies of dashboards where the data showed both severe and subtle equity issues in distribution of teaching and service between associate professors and other ranks and between women and men. Department members worked in groups to identify the equity issues and consider potential causes. We then presented a series of 12 organizational practices that might be used to address equity issues. The organizational practices were summarized in 2 page worksheets which outlined an equity issue a department was trying to solve, and provided example policy or practice language for how to address it. We also collected real workload policy examples from STEM and Social Science departments across the U.S. and created a policy workbook. We provided this policy workbook to participating departments. Department teams then considered what equity issues they saw from their own data and/or wanted to proactively design for, and which organizational practices and/or policies they wanted to put in place. Finally, they completed a two-three page Department Equity Action Plan with concrete plans for reform of current workload policies and/or creation of new practices and policies and brought these proposals back to their departments for approval. The goal of this intervention was long term change in the choice environment of department divisions of labor (e.g. routine practices and conditions) as well as improved action readiness on the part of faculty and department administrators to use data to discuss equity issues and enact equity-minded solutions with colleagues.

The fourth part of the intervention was a 4 week individual time management and planning seminar for members of the academic departments. This activity was optional rather than required, unlike the other 3 interventions. We ran the same workshop twice with a total of 36 members from 9 of the 17 departments. The workshop was conducted via 4 online webinars, and readings and homework completed in between sessions. The focus of the first workshop was introducing participants to Toggl, the online time diary service, and recommending five strategies for aligning time and priorities as supported by the literature. In the second workshop, participants reflected on their time records, discussed their time saboteurs, and subsequently identified at least one time use strategy to put in place over the next three weeks. In the third workshop, participants examined their new work activity requests and discussed the importance of saying yes and no strategically as well as strategies for saying no to requests. The fourth workshop focused on challenges regarding implementing time strategies and how to change habits based on the relevant research. The webinars were delivered by the authors drawing on a synthesis of time management strategies. There was also a homework component completed between sessions. Studies of time use have found large discrepancies between individuals’ estimates of their time use and actual use [5]. We used a time diary approach developed and implemented by in previous research [6] to engage participants in recording new work requests as they came in, their responses to the new work requests and reasons for their responses and produced a report for each participant at the end of 4 weeks. We also provided training for participants on how to use Toggl to record their work activities and time weekly which allows for a weekly report to be published. Concrete strategies provided included prioritizing work goals, creating a network of colleagues to help gauge alignment of new requests with goals, timing and practicing “nos,” and measuring success quarterly regarding responses to requests and outcomes. This intervention provided department participants with an opportunity for reflection on their career priorities, time use and negotiation strategies with colleagues.

**Survey design and variables**

We designed a cross sectional survey to examine our three research questions, compare control and participating departments to each other, and understand the influence of the 4 part intervention in participating departments over time. An expert panel with content knowledge and survey design experience reviewed the survey instrument and provided suggestions for revision, which we used to improve our items and constructs. We attained content, construct and face validity, appropriateness, sensibility, and relevance of the test and its items through this process [7].

Five constructs were identified through the literature and expert review. We created composite scales of department work practices, department conditions, action readiness, faculty outcomes, and satisfaction with teaching and service activities (classes, advising, and committees). The *department work practices* construct captures the presence of organizational practices that create transparency like the creation of dashboards, rotations of time intensive roles, differentiated workloads, and revisions of merit policies. The *department work conditions* construct measures the extent to which faculty agreed about their department’s understanding of implicit bias, and whether there are conditions existing as backdrops and contexts to facilitate fair workload such as perceptions of transparency, clear benchmarks, rewards systems and roles. *Action readiness* is operationalized as the extent to which department faculty were confident about using data to create equitable workloads, allocating resources more equitably, and creating more transparent benchmarks. The *faculty outcomes* construct measured the perception of fairness as it related to workload allocation, and intent to leave the University. The *teaching and service workload satisfaction* construct included satisfaction with the process of assigning and amount of work involved in classes, advising and committees.

These items were measured using a 5-point Likert-type response scale (e.g. 1-very dissatisfied, 2-dissatisfied, 3-neithed satisfied nor dissatisfied, 4-satisfied, 5-very satisfied), and the mean of the items was used as the overall measure of the constructs. The full list of survey questions that contributed to each construct is provided in S3 Table.

**Faculty demographics and department characteristics**

We analyzed the variables of gender (man = 0, woman = 1), race (White = 0, Faculty of Color = 1), and rank (dummy coded with assistant professors as the referent group). In S1 Table we provide descriptive statistics for the survey items and the final constructs for all post-survey respondents; in S2 Table we provide descriptive information about sample demographics.

**The participants**

The Researchers received 30 applications from 16 institutions. Of the 16 institutions, one was a baccalaureate institution, 6 master’s, and 9 doctoral/research institutions. All departments completed the pre-survey. Pre-survey invitations were sent out to 658 faculty. Of 658 invited faculty, 70.5% (n=464) responded to the pre-survey.

**Sample matching**

Once pre-survey data collection was complete, we sorted applications into an eligible pool of matched departments. We engaged in narrow stratified random sampling. We matched departments that applied on several criteria shown in prior research to impact previous diversity and academic focused interventions. We matched participating and control departments based on four categories: institution type (research/doctoral, comprehensive/MA, baccalaureate), discipline (natural science/social science), gender composition (low 1-34%, medium 35-50%, high 51-100%), and department size (small 0-15, medium 16-30, and large 31-60). We matched as many as possible on four or three categories; two departments could only be matched on two categories; we prioritized STEM and gender composition over institution type and department size in the matching process.

- Discipline: STEM (ENGR and CMNS math, computer science, physics, chemistry), Social Science, and Natural Science
- Institutional type (research/doctoral; comprehensive/MA; baccalaureate)
- Gender composition/percentage of women faculty, low 1-34%, medium 35-50%, high 51-100%
- Department size (small 0-15, medium 16-30, and large 31-60)

**Intervention phase**

Departments were sent a letter notifying them of their status in the project as a participating or control department. We worked with the 17 participating departments for 18 months on the four interventions noted above. Researchers had no contact at all with control departments after the pre-survey was competed. The 17 participating departments were asked to keep all project materials within their department and not share outside their department or on websites. In some cases, participating and control departments resided in the same institutions; however every attempt was made to keep project materials and efforts sequestered within the participating departments.

**Post survey**

At the completion of the project, 18 months after it began, we sent a post-survey to control and participating departments. We sent 635 post-survey invitations to department faculty and 472 agreed to participate. As is common when testing interventions over a significant period of time (e.g. 18 months) there were some members of departments that had retired or left the department, and some new faculty who had not taken the pre-survey. In addition, the post-survey response rate was 74.3; we matched 326 participants (69% of all respondents) from control and participating departments who took both pre and post surveys. However, given the primary focus in understanding the overall work practices, conditions, and action readiness in participating departments pre and post, and overall comparison of participating post-survey to control survey, the data was appropriate to answer our research questions.

**Data analysis**

We tested the construct validity of each latent factor using Confirmatory Factor Analysis (CFA) [8]. In this step we determined standardized item loadings on each latent factor (S1 Table). All items had standardized item loadings above 0.5. Given that CFA allows exploring and confirming the originally envisioned theoretical structure, we retained items based on standardized item loadings, conceptual value of the item to the construct, and the final fit of the model. The final CFA model resulted in good model fit indices: RMSEA=.058 and CFI=.925. Comparative Fit Index (CFI) values of 0.90 and higher indicate an excellent model fit; Root Mean Square Error of Approximation (RMSEA) values of 0.06 and lower are typically considered appropriate [9].

Second, we conducted one-way ANOVA analyses to determine whether there were significant differences in key constructs of department work practices, department conditions, action readiness and satisfaction with teaching and service workload based on gender, race, and rank. Third, we ran regression analyses for faculty outcomes on the key factors of department work practices, department conditions, action readiness, and satisfaction with teaching and service activities for participating faculty in pre- and post-surveys, controlling for gender, race, and rank. Then we ran (a) *t*-test analyses on individual items for all pre- and post-survey respondents to determine differences between participating and control faculty, (b) and regression analyses on individual items for all post-survey respondents to determine the effect of participating in project activities, controlling for gender, race, and rank.

Next, paired samples t-tests were calculated to determine whether there were significant mean differences between matched respondents’ answers to pre- and post-surveys. Effect sizes were calculated using Cohen’s d. Finally, we ran regression analyses on change scores in individual items and constructs to determine significant differences in matched respondents’ answers to pre- and post-surveys based on their participation in project activities (see S4 Table).

**Limitations**

There are several limitations to this research design. First, when it was time to assign the 30 departments as matched pairs, we were not able to create a clean split of 15/15 departments. Because the interventions would need to occur three out of four times in face to face meetings, simultaneously in 3 states of NC, MD, and MA, we needed to make sure there were enough participating institutions in each state to warrant a group being formed there. As a result we created 18 matched pairs, and then randomly assigned one department from each pair to the participating or control group.

Although we studied matched pairs, the intent of the study was to understand the departments as two larger groups—those who participated as a group and those who served as control departments as a group. Ideally, we would match each participant in control and treatment groups perfectly, without anyone leaving the study during the intervention. Yet, this is not possible outside of a laboratory, and we were more interested in understanding faculty experience of the changed choice environment.

Second, not all departments engaged in all activities at exactly the same level or intensity. We ran the interventions in three states (North Carolina, Maryland and Massachusetts) with identical project materials, requirements, and deadlines for all departments. Team sizes ranged from three to five members. All departments engaged in the three required interventions (e.g. implicit bias workshop, creation of dashboard, review of equity and implementation of equity-minded policies and practices). The time management professional development seminar was available to members from all departments but not all departments engaged. The nature of the project as a human endeavor over 18 months makes this variation inevitable. We were also unable to match control and experiment departments by department chair’s gender, and thus do not know the influence of this variable. There is also previous research that suggests that the priority a department puts on diversity, and in general faculty interest in reform can shape intervention outcomes [10,11]. The gender of the department chair and general willingness of department faculty are factor for which we could not control, and thus may have had some influence on the outcomes.

**References**

1. Carnes M, Devine PG, Isaac C, Manwell LB, Ford CE, Byars-Winston A, et al. Promoting institutional change through bias literacy. Journal of Diversity in Higher Education 2012; 5: 63–77.

2. Devine PG, Forscher PS, Cox WT, Kaatz A, Sheridan J, Carnes M. A gender bias habit-breaking intervention led to increased hiring of female faculty in STEMM departments. Journal of experimental social psychology 2017; 73:211-5.

3. Moss-Racusin CA, van der Toorn J, Dovidio JF, Brescoll VL, Graham MJ, Handelsman J. Scientific diversity interventions. Science 2014; 343(6171): 615-616

4. Shields SA. Website for WAGES (Workshop Activity for Gender Equity Simulation), a National Science Foundation-funded project. 2009 [cited 5 November 2018]. Available from: <http://wages.la.psu.edu/>

5. Sullivan O, Gershuny J. Cross‐national changes in time‐use: some sociological (hi) stories re‐examined. The British journal of sociology. 2001; 52(2):331-47.

6. O’Meara K, Kuvaeva A, Nyunt G, Waugaman C, Jackson R. Asked more often: Gender differences in faculty workload in research universities and the work interactions that shape them. American Educational Research Journal 2017; 54(6):1154-86.

7. Holden RR. Face validity. The corsini encyclopedia of psychology. 2010;:1-2.

8. Hancock, GR, and Ralph O. Mueller, eds. Structural equation modeling: A second course. Charlotte: Information Age Publishing; 2013.

9. Hu LT, Bentler PM. Cutoff criteria for fit indexes in covariance structure analysis: Conventional criteria versus new alternatives. Structural equation modeling: a multidisciplinary journal. 1999; 6(1):1-55.

10. Moss-Racusin CA, van der Toorn J, Dovidio JF, Brescoll VL, Graham MJ, Handelsman J. A “scientific diversity” intervention to reduce gender bias in a sample of life scientists. CBE-Life Sciences Education 2016; 15(3): 15:ar29,1- 15:ar29, 11.

11. Su X, Bozeman B. Family friendly policies in STEM departments: Awareness and determinants. Research in Higher Education 2016; 57(8): 990-1009.
