# Supplementary material for: Undoing disparities in faculty workloads: A randomized trial experiment
Source: PLoS One. 2018 Dec 19;13(12):e0207316. doi: 10.1371/journal.pone.0207316 (PMC6300212; doi:10.1371/journal.pone.0207316)
Supplement: S1 Table — (DOCX) [file pone.0207316.s002.docx]

**S1 Table. Survey Items Descriptive Statistics, All Post-Survey Respondents**

| Constructs | Survey Item | Mean | Std.  Dev. | Standardized  item loading |
| --- | --- | --- | --- | --- |
| Department Work Practices* | Transparent information about faculty work activities for all department faculty to see (e.g., number of advisees, committees, size of classes). | 2.04 | .85 | .749 |
|  | Transparent information about compensation for key roles (e.g., the agreed upon overload or support for taking on specific administrative roles). | 1.87 | .88 | .717 |
|  | Planned rotations of time intensive campus service or administrative roles. | 1.72 | .86 | .755 |
|  | Credit systems that allow faculty doing more than their share in one area to receive credit to do less in another area. | 1.71 | .89 | .823 |
|  | Differentiated workloads wherein faculty can adjust the percent of their effort across teaching, research and campus service roles. | 1.91 | .95 | .682 |
|  | Revision of shared governance rules to ensure that there are not more faculty on committees than are needed to get work done. | 1.60 | .80 | .772 |
|  | Revision of department reward system documents (e.g., merit, annual review or promotion and tenure criteria as appropriate) to include more credit for teaching and campus service activities. | 1.76 | .87 | .718 |
|  | Revision of department reward system documents (e.g., merit, annual review or promotion and tenure criteria as appropriate) to differentiate serving on committees versus providing leadership for them. | 1.90 | .89 | .766 |
| Department Conditions** | Faculty in our department have a good understanding of unconscious bias and how it shapes faculty workload. | 2.04 | .80 | .599 |
|  | Our department has consensus on a clear set of priorities for faculty time. | 1.97 | .91 | .757 |
|  | I think most people in our department feel work is distributed fairly. | 2.14 | .84 | .714 |
|  | There is a strong commitment within our department faculty that workload be fair. | 2.48 | .75 | .810 |
|  | Our workload decisions tend to be informed by data that is visible and widely available to everyone. | 1.96 | .90 | .760 |
|  | There are clearly identified benchmarks for expected campus service contributions. | 1.72 | .85 | .724 |
|  | There are clear benchmarks for expected advising contributions. | 2.02 | .91 | .633 |
|  | There is transparency related to faculty workload (e.g., data about faculty teaching, mentoring, and campus service activities available for public scrutiny). | 2.06 | .91 | .750 |
|  | Our department chair and faculty have discussed and agreed upon which roles faculty will be compensated for (with additional resources), and which are simply part of their jobs. | 2.08 | .89 | .702 |
|  | The most important teaching, mentoring and campus service work I do is credited within my department reward system. | 2.30 | .86 | .706 |
|  | Our department reward system differentiates between membership and leadership in campus service. | 2.18 | .77 | .538 |
| Satisfaction with Classes | Number of classes taught | 3.87 | 1.13 | .880 |
|  | Class sizes | 3.75 | 1.05 | .661 |
|  | Support for classes (TAs, RAs) | 3.22 | 1.25 | .610 |
| Satisfaction with Class Assignments | The kinds of classes you teach | 4.27 | .88 | .735 |
|  | The process in which classes are assigned | 3.89 | 1.06 | .882 |
| Satisfaction with Advising | The number of advisees you have | 3.57 | 1.09 | .830 |
|  | The kinds of advisees you have (undergraduate, continuing education, graduate students) | 3.87 | .90 | .779 |
|  | The process in which advisees are assigned | 3.62 | 1.01 | .780 |
| Satisfaction with Committees | The number of committees on which you serve | 3.73 | .83 | .809 |
|  | The amount of work you do on committees versus the amount others do | 3.45 | 1.01 | .777 |
|  | The attractiveness (e.g., value, visibility, importance, personal preference) of the committees on which you serve | 3.54 | .88 | .706 |
|  | The process in which committee assignments are made | 3.45 | .96 | .834 |
|  | The number of committees you chair | 3.69 | .78 | .679 |
| Satisfaction with Department Interactions and Negotiations over Time and Work Activities | The percent of time you spend each semester on research and/or professional development. | 3.00 | 1.16 | .725 |
|  | The percent of time you spend each week on work that is meaningful to you. | 3.34 | 1.11 | .763 |
|  | Your ability to say no to additional requests from colleagues on campus without harming your career prospects | 3.35 | 1.05 | .777 |
|  | I feel comfortable protecting time in my schedule for research and/or professional development. | 3.86 | 1.37 | .695 |
|  | I feel comfortable asking for additional resources when being asked to take on a new and time-consuming task for the department. | 3.70 | 1.37 | .769 |
| Action Readiness | I know strategies I can use to improve the perception and reality of fairness in how work is assigned, taken up and rewarded in our department. | 3.60 | 1.28 | .850 |
|  | I have identified several concrete steps I can take to ensure greater equity in our department workload. | 3.37 | 1.32 | .827 |
|  | Use data to initiate a dialogue within my department about putting practices in place to ensure the teaching and campus service burden is shared by all. | 2.92 | 1.26 | .798 |
|  | Ask someone in my department who typically shirks department work to participate for the good of the order. | 2.27 | 1.30 | .719 |
|  | Work with colleagues to create more transparent benchmarks for all faculty for things like advising loads and committee assignments. | 3.11 | 1.23 | .911 |
|  | Work with colleagues to make sure resource allocation and the selection of individuals for any coveted positions are more transparent and intentional (an open and consistent process for everyone, not secret or case by case). | 3.00 | 1.26 | .926 |

* Department Work Practices: Please check which of the following policies or practices your department has in place and to what extent they are in place.

** Department Conditions: Please note the degree to which you agree or disagree with the following statements.
