# Supplementary material for: Undoing disparities in faculty workloads: A randomized trial experiment
Source: PLoS One. 2018 Dec 19;13(12):e0207316. doi: 10.1371/journal.pone.0207316 (PMC6300212; doi:10.1371/journal.pone.0207316)
Supplement: S2 Table — (DOCX) [file pone.0207316.s003.docx]

**S2 Table. Respondent Demographics, 2018**

| Rank | Assistant Professors | 21.2% |
| --- | --- | --- |
|  | Associate Professors | 29.8% |
|  | Full Professors | 28.9% |
|  | Non Tenure-Track Lecturer | 14.6% |
|  | Non Tenure-Track Researcher | 0.2% |
|  | Other | 5.3% |
|  | American Indian or Alaska Native | 0.7% |
| Race | Asian | 9.6% |
|  | Black/African American | 10.7% |
|  | White | 75.1% |
|  | Multi-Racial | 3.9% |
| Gender | Female | 45.8% |
|  | Male | 52.5% |
|  | Other | 1.8% |
