# Supplementary material for: Undoing disparities in faculty workloads: A randomized trial experiment
Source: PLoS One. 2018 Dec 19;13(12):e0207316. doi: 10.1371/journal.pone.0207316 (PMC6300212; doi:10.1371/journal.pone.0207316)
Supplement: S4 Table — (DOCX) [file pone.0207316.s005.docx]

**S4 Table. Regression Results on Change Scores for Participating vs. Control Departments, Pre- to Post- Matched Respondents**

| Constructs | Survey Item | Standardized regression coefficients |
| --- | --- | --- |
| Department Work Practices | Transparent information about faculty work activities for all department faculty to see (e.g., number of advisees, committees, size of classes). | .138* |
| Department Conditions | Faculty in our department have a good understanding of unconscious bias and how it shapes faculty workload. | .142* |
| Satisfaction with Classes | Number of classes taught | .140* |
| Satisfaction with Advising | The kinds of advisees you have (undergraduate, continuing education, graduate students) | .129* |
| Satisfaction with Committees | The number of committees on which you serve | .201** |
|  | The amount of work you do on committees versus the amount others do | .154** |
|  | The attractiveness (e.g., value, visibility, importance, personal preference) of the committees on which you serve | .275*** |
|  | The process in which committee assignments are made | .182** |
| Satisfaction with Department Interactions and Negotiations over Time and Work Activities | Your ability to say no to additional requests from colleagues on campus without harming your career prospects | .189** |
|  | I feel comfortable asking for additional resources when being asked to take on a new and time-consuming task for the department. | .133* |
| Action Readiness | I know strategies I can use to improve the perception and reality of fairness in how work is assigned, taken up and rewarded in our department. | .165** |
|  | I have identified several concrete steps I can take to ensure greater equity in our department workload. | .181** |
|  | Use data to initiate a dialogue within my department about putting practices in place to ensure the teaching and campus service burden is shared by all. | .251*** |
|  | Work with colleagues to create more transparent benchmarks for all faculty for things like advising loads and committee assignments. | .142* |
|  | Work with colleagues to make sure resource allocation and the selection of individuals for any coveted positions are more transparent and intentional (an open and consistent process for everyone, not secret or case by case). | .198** |
| Fair distribution of workload | Overall, do you feel the distribution of teaching and service work in your department is fair? | .228*** |

Regression analysis was performed on survey items controlling for gender, race, and rank. Significant items for participating faculty at **p* < .05. ***p* < .01. ****p* < .001.
